# Transient Abnormalities in Masking Tuning Curve in Early Progressive Hearing Loss Mouse Model

**DOI:** 10.1155/2018/6280969

**Published:** 2018-02-13

**Authors:** Marion Souchal, Ludimila Labanca, Sirley Alves da Silva Carvalho, Luciana Macedo de Resende, Christelle Blavignac, Paul Avan, Fabrice Giraudet

**Affiliations:** ^1^Neurosensory Biophysics, INSERM UMR 1107, Clermont Auvergne University, Clermont-Ferrand, France; ^2^Speech Therapy and Audiology Department, Federal University of Minas Gerais, Belo Horizonte, MG, Brazil; ^3^Cellular Health Imaging Center, Clermont Auvergne University, Clermont-Ferrand, France

## Abstract

Damage to cochlear outer hair cells (OHCs) usually affects frequency selectivity in proportion to hearing threshold increase. However, the current clinical heuristics that attributes poor hearing performance despite near-normal auditory sensitivity to auditory neuropathy or “hidden” synaptopathy overlooks possible underlying OHC impairment. Here, we document the part played by OHCs in influencing suprathreshold auditory performance in the presence of noise in a mouse model of progressive hair cell degeneration, the CD1 strain, at postnatal day 18–30 stages when high-frequency auditory thresholds remained near-normal. Nonetheless, total loss of high-frequency distortion product otoacoustic emissions pointed to nonfunctioning basal OHCs. This “discordant profile” came with a huge low-frequency shift of masking tuning curves that plot the level of interfering sound necessary to mask the response to a probe tone, against interfering frequency. Histology revealed intense OHC hair bundle abnormalities in the basal cochlea uncharacteristically associated with OHC survival and preserved coupling with the tectorial membrane. This pattern dismisses the superficial diagnosis of “hidden” neuropathy while underpinning a disorganization of cochlear frequency mapping with optimistic high-frequency auditory thresholds perhaps because responses to high frequencies are apically shifted. The audiometric advantage of frequency transposition is offset by enhanced masking by low-frequency sounds, a finding essential for guiding rehabilitation.

## 1. Introduction

Sensorineural hearing losses (SNHL) stem from a wide spectrum of diseases affecting the sensory receptors, outer hair cells (OHCs), inner hair cells (IHCs), or auditory neurons (afferent and even efferent fibers) [1–5]. Pure-tone audiometry is the routine clinical audiological test used for measuring hearing sensitivity, and the audiometric classification of hearing impairments is the main basis upon which audiologists determine their rehabilitation choice. Even though it cannot provide any fine-grained reflection of the mechanism of SNHL and may not delineate individual needs, it works in a large majority of cases because it is the damage to OHCs that usually accounts for the hearing impairment, particularly frequency selectivity that, in simple cases, is affected in proportion to the increase in hearing thresholds [6]. However, it is acknowledged that there are “discordant patterns,” that is, subjects with near-normal audiometric thresholds yet difficulties in speech intelligibility, especially in noisy environments [7], Their investigation has led to the discovery of auditory disorders that widely differ from the typical OHC-related SNHL, namely, auditory neuropathies and synaptopathies [8, 9]. Auditory neuropathies are revealed by abnormal auditory-evoked potentials although a recent picture has been substantiated in animal models of short overexposure to intense sounds, which develop noise-induced synaptopathies with no detectable auditory-evoked-potential abnormality [10–12]. In these conditions, pure-tone audiometry is obviously inadequate for predicting suprathreshold auditory perception tasks [13], as discrepancies among metrics of auditory performance are expected when SNHL arises, not from micromechanical stages but from transduction and action-potential generation or conduction. But pure-tone audiometry may also fail to provide a coherent picture of auditory performance in the case of pure OHC dysfunction, as shown by a mutation in the Nherf1 gene expressed only in OHCs at mature stages [14]. The mild ABR hearing threshold elevation of* Nherf1*^−/−^ mice at high frequencies is contradicted by an absence of high-frequency DPOAEs and by an inordinate sensitivity of mid/high frequencies to low-frequency maskers. This nonconventional functional pattern comes with peculiar OHC hair bundle shape anomalies in the basal part of the cochlea. Thus, the finding of inconsistencies between auditory thresholds and suprathreshold auditory performance, for example, frequency selectivity, cannot guarantee that these inconsistencies point to auditory neuropathy, a diagnosis that, even when neuropathy is “hidden,” might prompt clinicians to the prescription of auditory-neuropathy-specific intervention. Along the same line, recent papers stress the importance of OHC function as a determinant of speech-in-noise performance, highlighted by its decrease with decreased OAEs in a sample of subjects with audiometric thresholds within the normal range [15, 16]. All these studies warn against considering that hidden hearing loss excludes OHCs as a potential contributor.

Therefore, the goal of the present work is to better document the part played by OHCs in influencing suprathreshold auditory function. To bridge the gap between transgenic or knockout mouse models with precisely targeted deficits and human data in subjects for whom the causes of SNHL are difficult to track with unknown combinations of genetics, aging, and exposure to environmental factors, we sought to investigate a rapidly progressive sensorineural auditory impairment in a strain of noninbred mice, CD1 mice. These mice are known to exhibit early onset of hearing loss due to hair cell degeneration and to offer, between around 3 and 8 weeks after birth, a broad range of frequency intervals of hearing loss and proportion of damaged OHCs [17–19].

We performed a longitudinal functional study in CD1 mice at the first 30 postnatal days and observed progressive hearing loss, yet with a discrepancy between high-frequency audiometric thresholds, close to normal, and absence of DPOAE, akin to that described by Kamiya et al. [14] yet likely much more widespread as it results from generic OHC hair bundle abnormalities, not dependent on a very rare gene mutation. Masking tuning curves were built to test basal OHC functionality and scanning electron microscopy, to study OHC hair bundle shape anomalies in the basal cochlea. Observations suggested a transient disorganization of the cochlear frequency mapping.

## 2. Material and Methods

### 2.1. Animals

Male CD1 mice (*n* = 15) from Janvier Labs were included in this study at 18 days of age. The animals were maintained in temperature and humidity-controlled facilities. Ambient sound pressure levels inside the cages were below 40 dB SPL. For all hearing test experiments, mice were anesthetized with ketamine (100 mg/kg, i.p.) and xylazine (20 mg/kg, i.p.). Body temperature was maintained at 37°C (Microprobe Thermometer, BAT-12, WPI) with an isothermal pad (Homeothermic Blanket System, Harvard Apparatus). Prior to testing and to exclude middle ear damage, an otoscopic examination (using a binocular operating microscope) was performed on each mouse. Cochlear function was assessed via ABR and DPOAE at postnatal days 18, 21, 25, and 30 (i.e., P18, P21, P25, and P30). All the auditory tests were performed in a sound attenuated and electrically shielded recording chamber. After the final auditory test, animals were sacrificed for histological processing and scanning electron microscopy analyses of hair cell stereocilia. All procedures were approved by the Regional Ethics Committee for animal experiments in France (Comité d'Éthique pour l'Expérimentation Animale Auvergne; EC 92-12).

### 2.2. DPOAE Recording

All testing was conducted using a stimulation and acquisition chain (EPL Cochlear Function Test Suite, Eaton-Peabody Laboratories, Harvard Medical School) controlled by a computer (NI PXI-1031, National Instrument). A miniaturized acoustic system (consisting of two speakers and one microphone) was gently sealed into the ear canal. Using a dedicated software (EPL Cochlear Function Test Suite-Eaton, Peabody Laboratories, Harvard Medical School), the parameters used for the stimulation were *f*2/*f*1 = 1.20 with *L*1 = *L*2. DPOAEs were measured for *f*2 = 10, 15, 22, and 32 kHz, with *L*1 and *L*2 from 10 dB to 80 dB in steps of 5 dB. DPOAE threshold was defined as the lowest sound level producing a DPOAE with an amplitude of at least 7 dB above the noise floor. Absence of instrumental DPOAE was verified in dead mice from the same batch at each frequency.

### 2.3. ABR Thresholds

Responses were recorded with needle electrodes (stainless steel, diameter: 0.4 mm, Medtronic Xomed Inc.) inserted through the skin at the vertex (active electrode) and ipsilateral mastoid (negative electrode) and in the neck region (ground). All electrode impedances were similar and <5 kΩ at the start of the test. The responses from the electrodes were amplified (×100,000), filtered (100–3,000 Hz), digitally converted, and averaged (300 sweeps) by a two-channel recording system (Neuropack *μ*®-MEB 9104, Nihon Kohden). The acoustic stimulus was generated by an arbitrary waveform generator (TG4001, Thurlby Thandar Instruments) which produced tone-bursts at 5, 10, 15, 22, 27, and 32 kHz. The envelope of the tone-burst was built using the Blackman-Harris formula (with the whole stimulus window containing 60 periods of the tone), in order to remove the artefact impulse sound produced when an earphone receives a too steeply rising signal. The acoustic stimulus intensity was controlled by an attenuator (PA4/SM3/HB6/XB1, Tucker Davis Technologies); then it was sent to the high-frequency earphone in the external ear canal (Number 40–137 8 Ω 70 W 8A4, Radio Shack Japan). The intensity at which an ABR waveform was still visually detected above noise floor was defined as the ABR threshold.

### 2.4. Masking Tuning Curve (MTC)

The probe stimulus was the same for the measurement of ABR, emitted 10 dB above the ABR threshold, at the target frequencies of 10, 22, and 32 kHz. The masking sound was a pure tone generated by a second generator (TG4001, Thurlby Thandar Instruments) and sent by a separate electronic and acoustic track to avoid electric distortion. The intensity of the masking sound considered effective induced a reduction of 50% of the ABR wave I amplitude generated in response to the target stimulus. It was determined for different masking frequencies swept above and below the target frequency.

### 2.5. Electron Microscopy

The mice were deeply anesthetized with pentobarbital (50 mg/kg) and sacrificed by transcardiac perfusion with freshly prepared 4% PFA in 0.1 M PBS. The cochleas were removed from the temporal bones under a binocular microscope in PHEM buffer (Pipes, Hepes, EGTA, and Magnesium) and fixed in 2.5% glutaraldehyde overnight at 4°C. The next day, samples were dehydrated in a series of alcohol baths from 25° to 100° and then HMDS (Hexamethyldisilazane) and metalized to be visualized with a scanning electron microscope with a field emission gun (FEG) (JSM-6060LV, JEOL). A count of normal, altered, and missing OHC stereocilia bundles in the apical (corresponding to the place coding frequencies around 10 kHz), middle (place coding frequencies around 20 kHz), and basal regions (place coding frequencies around 32 kHz) at each postnatal age was achieved. Hair bundles were considered altered when they had asymmetrical, linear, or hooked shapes.

### 2.6. Data Analysis

Graphs represent either individual plots or means ± standard error of the mean (sem). Statistical analysis was performed using Shapiro-Wilk test (Sigmaplot®, Systat Software Inc.). Differences were considered statistically significant when *P* < 0.05.

## 3. Results

### 3.1. Progressive Age-Related Hearing Loss of CD1 Mice

#### 3.1.1. ABR Threshold Changes with Age

**Figure 1:**
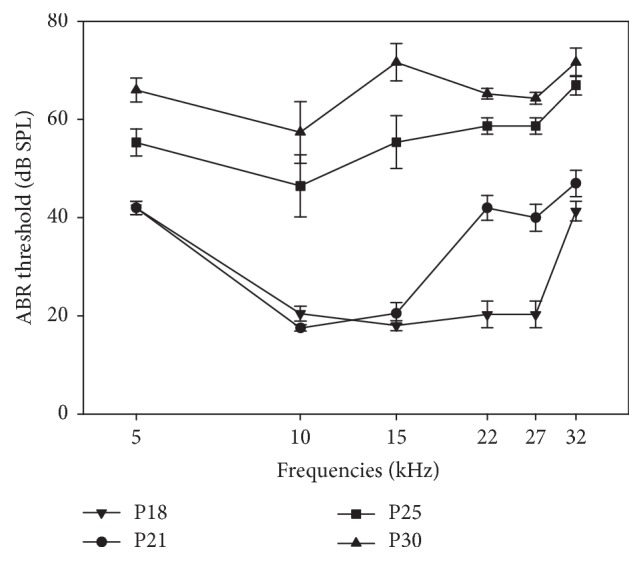
*ABR hearing thresholds of the CD1 mouse as a function of age*. Mean ABR hearing thresholds (±sem) measured for P18, P21, P25, and P30 CD1 mice (*n* = 15) with tone-bursts at 5, 10, 15, 22, 27, and 32 kHz.

We first assessed the hearing sensitivity in the 5 to 32 kHz frequency range. The changes in ABR hearing thresholds (mean ± sem) over time are illustrated in Figure 1. Hearing loss in CD1 mice was significant and progressive. A few days after the onset of hearing, on P18, mean ABR hearing thresholds are consistent with a normal hearing sensitivity at frequencies from 10 to 27 kHz but not at 32 kHz (41 ± 2 dB SPL). Three days later, the hearing loss had progressed substantially at 22 and 27 kHz, from normal hearing to mild hearing loss (increase of between 20 and 22 dB in average compared to P18). At P25, the hearing impairment was more pronounced for high frequencies from 22 to 32 kHz with ABR thresholds reaching 59 to 67 ± 2 dB SPL. For middle frequencies, the ABR hearing threshold increase was 35 dB for 15 kHz, 29 dB for 10 kHz, and 15 dB for 5 kHz. At 1 month, the ABR audiogram was flat at all frequencies tested with near 50 dB hearing loss.

So, there was a progressive elevation of the hearing threshold extending from high to low frequencies with a clear deterioration between P18 and P25. Between P25 and P30, the elevation of ABR thresholds slowed down.

#### 3.1.2. Progressive Impairment of DPOAE Growth Functions

**Figure 2:**
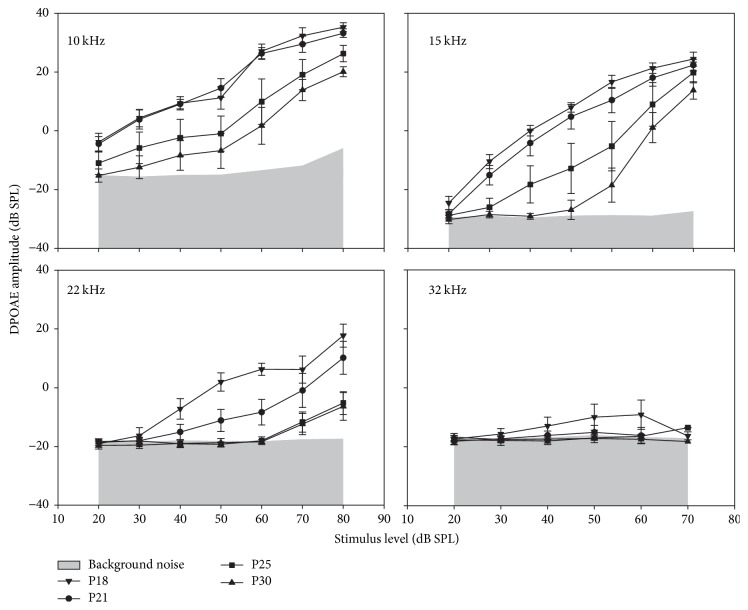
*DPOAE growth functions illustrating mean DPOAE amplitudes* (±sem) for primary levels ranging from 20 to 80 dB SPL in P18, P21, P25, and P30 CD1 mice at 10, 15, 22, and 32 kHz.

The mean DPOAE amplitudes as a function of the *f*2 primary level, that is, its growth functions, are shown in Figure 2 for 4 frequencies 10, 15, 22, and 32 kHz at P18, P21, P25, and P30. Except for the higher frequency (32 kHz) at P18, the general shape of the growth functions was characterized by a monotonically increasing DPOAE amplitude. At 32 kHz frequency, only P18 mice had detectable DPOAEs with very small amplitudes, with a signal-to-noise ratio of 7 (±5) for *L*2 = 60 dB. At all other frequencies, the growth functions shifted to the right more or less rapidly with increasing age. At P21 and lower frequencies 10 and 15 kHz, growth functions showed no significant change and only a little more than 10 dB downward shift at P25 and an additional 5 to 10 dB decrease at P30. Higher frequencies showed faster changes, −5 to −15 dB changes as soon as P21 at 22 kHz, while DPOAEs hardly emerged above noise at the highest stimulus levels after P25.

Relative to P18, ABR thresholds only increased by 30 to 40 dB at P25, suggesting the persistence of some degree of amplification by the cochlear amplifier, thought to normally have a 60 dB gain [20–23]. If OHCs are still functional enough to produce some gain, one might expect DPOAEs to persist at higher levels at least at high stimulus intensities. We therefore decided to look at individual cases assessed with the ABR and DPOAE in order to highlight the possible discrepancies between these two techniques.

### 3.2. Slight Increase in ABR Hearing Thresholds Contrasts with Defective Responses of the OHCs at the Base and Shifted Tips of Masking Tuning Curves

**Figure 3:**
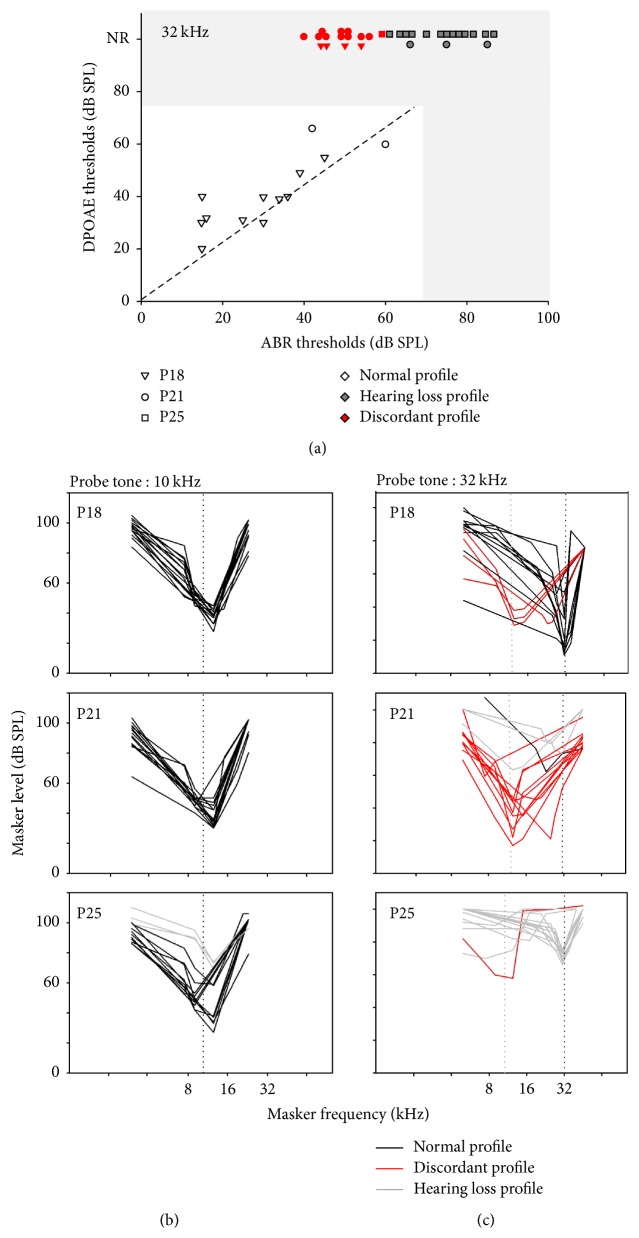
*Relationships between ABR and DPOAE thresholds and MTC at high frequencies*. Scatterplots of the individual DPOAE thresholds as a function of ABR thresholds at 32 kHz (a). Diagonal line: DPOAE and ABR thresholds are equal. Red symbols and red MTC correspond to individual with only slight increase in ABR thresholds (≤35 dB) but increase of DPOAE thresholds ≥40 dB or nonrecordable DPOAE (NR). The shaded areas correspond to the thresholds for which it is thought that OHCs have lost their function as they do not either generate gain, hence a 60 dB ABR threshold elevation, or emit distortion products. Individual masking tuning curves are presented for a probe tone at 10 kHz (b) and 32 kHz (c). Different symbols for different ages (see keys in (a)). Different lines for different MTC profiles (see keys in (c)).

The scatterplot of DPOAE thresholds at 32 kHz as a function of ABR thresholds and individual MTCs for a 32 kHz probe are represented in Figures 3(a) and 3(c), respectively. In some ears, the thresholds measured with these two techniques seemed well correlated, approximately along the diagonal line of the plot (Figure 3(a), dashed line). In view of these cases, a surprising discrepancy appears, with ears showing mild ABR threshold elevations yet no DPOAE (Figure 3(a), “discordant” points in red). The MTCs plotted in these cases at 32 kHz (Figure 3(c), red lines) contrast with the MTCs built in animals with a good correspondence between ABR and DPOAE thresholds (Figure 3(c), black lines) and with all MTCs built at 10 kHz, a frequency at which the animals kept normal thresholds between P18 and P25 (Figure 3(b)). More precisely, MTCs in black display a V-shaped profile with a deep tip at a frequency slightly above the probe frequency, corresponding to the most efficient frequency at which the masker interferes with the probe. At tip frequency, probe and masker levels are very close to each other. MTCs in red, in contrast, have no identifiable tip around the probe frequency, and the most efficient masker has a much lower frequency often lying around 12 kHz but sometimes between 16 and 32 kHz. A possible reading of these MTCs is that they only display a hypersensitive tail while the tip has become too shallow to be visible. The discordant profile, already observed at P18 (3 cases) occurred more frequently at P21 (10 cases) but almost vanished at P25 (one case, with most MTCs showing a blunt and elevated tip at the probe frequency).

**Figure 4:**
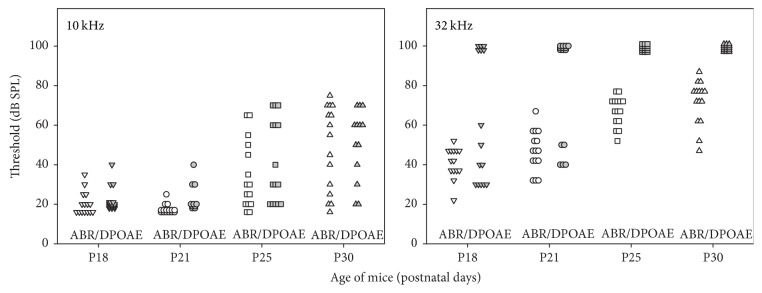
*ABR thresholds and DPOAE thresholds as a function of age*. Individual ABR hearing thresholds and individual DPOAE thresholds measured for P18, P21, P25, and P30 CD1 mice (*n* = 15, different symbols at different ages) at 10 and 32 kHz.

The distributions of ABR and DPOAE thresholds as a function of age at 10 and 32 kHz differ in the following manner (Figure 4). At 10 kHz, DPOAE and ABR thresholds tended to covary. They were normal (≤40 dB SPL) and not much scattered at P18 and P21. At P25 and P30, both thresholds were more scattered and tended to increase by similar degrees, from 20 up to 75 dB SPL. This is not what happened at 32 kHz. At P18, despite ABR thresholds not exceeding 55 dB SPL, 6 mice already had lost their DPOAEs. The difference between ABR and DPOAE thresholds (when DPOAEs were still present) could reach 40 dB. At increasing ages, the ABR thresholds gradually shifted upwards. The discrepancy between ABRs and DPOAEs was still present with no DPOAE in 10 mice at P21 and no DPOAE in all mice at P25 and P30.

**Figure 5:**
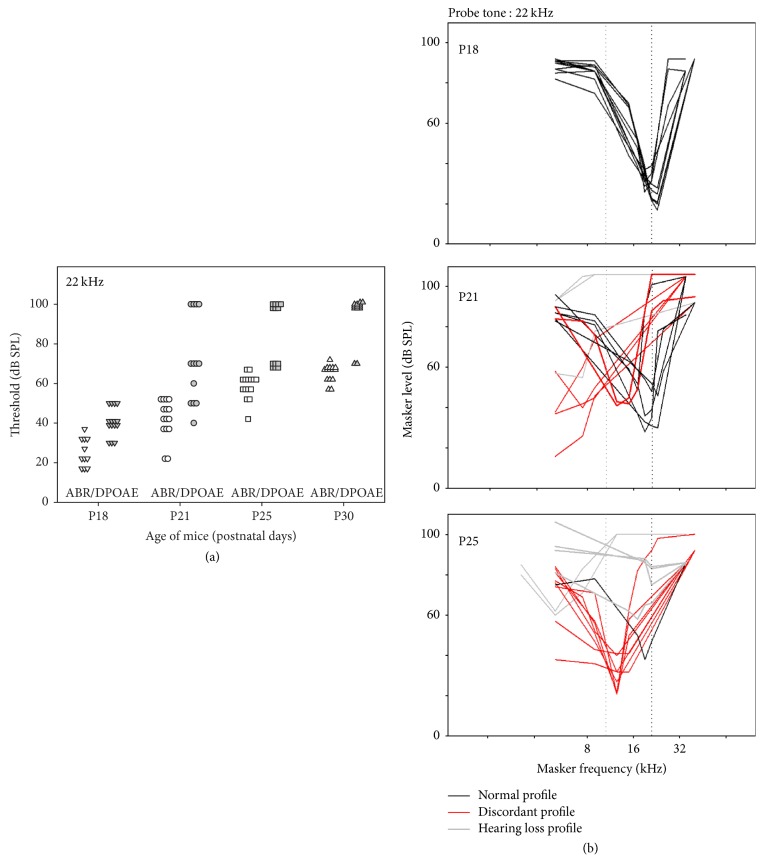
*Distribution of ABR hearing thresholds and DPOAE thresholds and MTC at 22 kHz*. Individual ABR hearing thresholds and individual DPOAE thresholds measured for P18, P21, P25, and P30 CD1 mice (*n* = 15, different symbols at different ages) at 22 kHz (a). Individual masking tuning curves for a probe tone at 22 kHz (b).

At the intermediate frequency 22 kHz, ABR thresholds and DPOAE thresholds were still similar at P18 with corresponding MTC tips near the probe frequency (Figures 5(a) and 5(b)). At P21, 5 mice had no DPOAE despite only mildly elevated ABR thresholds, <40 dB SPL for some individuals (normal around 20 dB SPL). For those mice, the most efficient masking shifted towards lower frequencies (Figure 5(b), red curves). At P25, 8 mice presented a discordant profile similar to that previously described at 32 kHz and in 6 mice no MTC tip or tail could be identified (Figures 5(a) and 5(b)). Among the mice with discordant profile, some animals still had residual DPOAEs yet with a threshold near 70 dB SPL.

**Figure 6:**
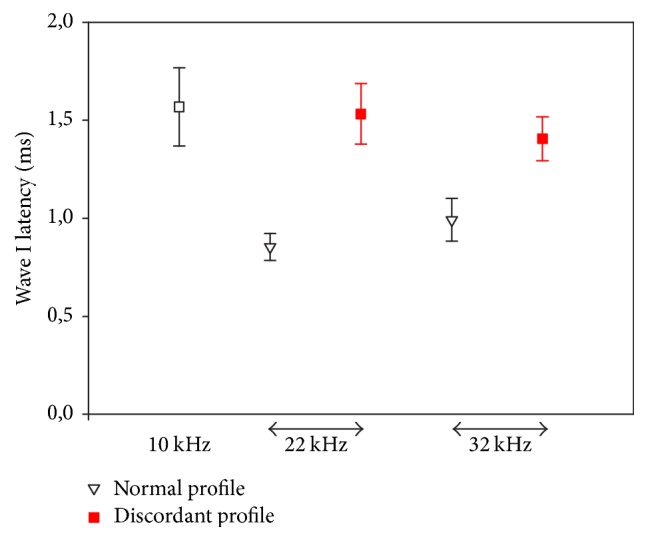
*ABR wave I latency* at 10, 22, and 32 kHz for a stimulus sound level of 40 dB SPL in mice presenting normal and discordant profiles.

In all mice, ABR wave I latencies in response to a 10 kHz probe were around 1.5 ms for a stimulus at 40 dB SPL. At 22 and 32 kHz, for normal mice, they were, respectively, 0.85 and 1 ms (Figure 6). The difference in ABR wave I latencies at this sound level between 10 kHz and 22 and 32 kHz was consistent with the base-to-apex cochlea frequency map. In mice with discordant profiles, wave I latency at 22 and 32 kHz was near 1.5 ms, thus in the same range for 10 kHz tone-bursts.

### 3.3. Morphological Features Observed with Scanning Electron Microscope (SEM)

**Figure 7:**
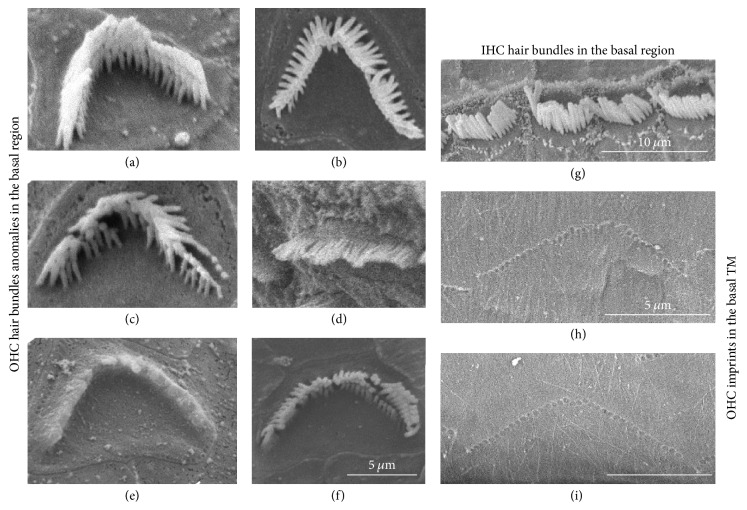
*Abnormal hair bundle shapes observed in the basal cochlear region*. SEM pictures of organ of Corti sections ((a)–(g)) and of tectorial membrane (TM) ((h), (i)) from CD1 mice. (a) Normal OHC hair bundle. (b) Asymmetrical hair bundle. (c) OHC with damaged stereocilia. (d) Linear OHC hair bundles. (e) OHC with fused stereocilia. (f) Hook-shaped OHC hair bundle. (g) Normal appearance of IHCs. ((h), (i)) Imprints left by abnormal (h) and normal (i) OHC stereocilia bundles in the TM in the basal region of cochlea.

**Figure 8:**
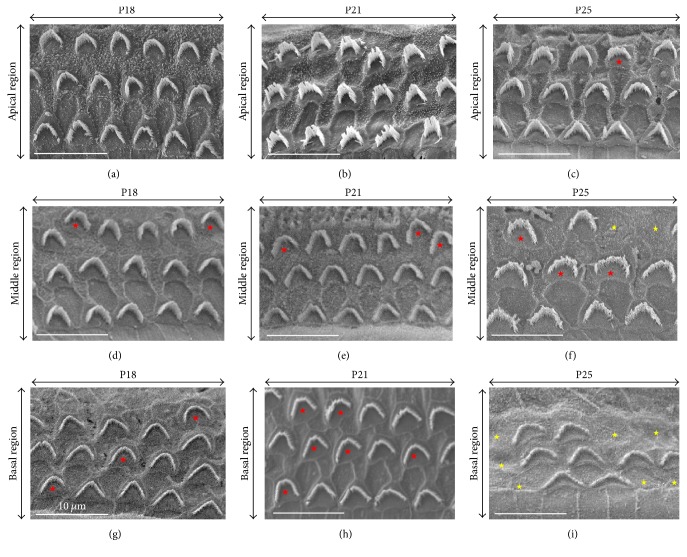
*Abnormal OHC hair bundle shapes observed at the cochlear base in CD1 mice*. SEM pictures of organ of Corti sections from CD1 mice at P18 ((a), (d), and (g)), P21 ((b), (e), and (h)), and P25 ((c), (f), and (i)). ((a), (b)) In the apical region, the OHC stereocilia bundles are normal at P18 and P21. (c) This apical section contains one OHC with an abnormal linear shape (red star). ((d), (e), and (f)) In the middle region at P18, P21, and P25, abnormal stereocilia bundles are found (asymmetric, linear, or hook-shaped) (red stars), and a few OHCs are missing at P25 (yellow stars). ((g), (h), and (i)) In the basal region, abnormal stereocilia bundles are observed at P18 and P21 (red stars, (g), (h)) and OHC losses are seen at P25 (yellow stars, (i)).

We next looked at the OHC hair bundle aspect in different regions of the cochlea in CD1 mice (Figures 7 and 8). SEM analysis of the middle and basal regions of the cochlea showed hair bundle anomalies in OHCs but not IHCs (Figures 7(a)–7(g)). Some OHC hair bundles in this region displayed altered, asymmetrical, linear, or hooked shapes (Figures 7(a)–7(f)). In the basal region, OHC hair bundles remained anchored in the tectorial membrane (TM) by their taller raw of stereocilia even when bundles had abnormal shapes (Figures 7(h) and 7(i)).

**Figure 9:**
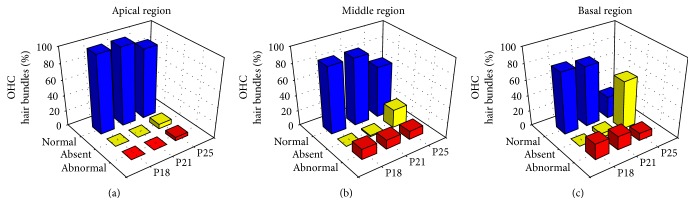
*The percentage of abnormal stereocilia bundles is greater at the base of the CD1 cochlea*. Amount of normal, abnormal, and absent OHC stereocilia bundles for P18 (*N* = 4), P21 (*N* = 6), and P25 (*N* = 4) CD1 mice in the apical, middle, and basal region.

A count of normal, altered, and missing OHC stereocilia bundles for each group of mice in the apical, middle, and basal region of the cochlea was achieved, and the results are shown in Figure 9. CD1-related OHC abnormalities are most evident in the basal region, with abnormal hair bundles in about 20% of cells from the earliest stage (Figure 8, red stars; Figure 9(c)). After P21, the main defect is the large percentage of missing OHCs, up to 62% (Figure 8, yellow stars). In the middle region, the progression of OHC abnormalities is qualitatively similar yet milder, with about 10% abnormally shaped hair bundles, stable during the period of interest, and only 23% missing OHCs at P25 (Figure 8; Figure 9(b)). The apical part of the CD1 cochlea is almost immune from defects at P18 and P21 (Figures 8(a), 8(b), and 8(c); Figure 9(a)), and, only at P25, 5 and 6% of OHCs show disorganized bundles or are absent.

## 4. Discussion

In CD1 mice, progressive hearing loss, already present at high frequencies at P18, rapidly extends to lower frequencies by P25 as already reported [17–19]. The prominent contribution of OHCs to this hearing loss was documented using microscopy, whereas the well-acknowledged damage to afferent neurons coexisting with that affecting hair cells was not the focus of this study. The variability in the degrees and progression of respective damage to these structures, also well-accepted, is viewed as supporting the CD1 model as a suitable though accelerated model for human presbycusis that shares similar characteristics [24].

In some mice, hearing loss combined an increase in ABR thresholds with a concomitant increase in DPOAE detection thresholds at the same frequencies, accompanied by a gradual loss of OHCs. However, a subgroup of mice displayed discrepancies, at high frequencies, between elevated DPOAE thresholds and less affected ABR thresholds. When DPOAEs become undetectable (i.e., thresholds well above 70 dB SPL), this indicates a loss of OHC function in the basal cochlea, which is not consistent with the small elevation (≤35 dB) of ABR thresholds from 22 kHz up. The OHCs, a key element of the cochlear amplifier, are thought to increase cochlear sensitivity by 50–60 dB [20–23]. Complete loss of OHC function signaled by complete loss of DPOAEs should therefore lead to an increase in hearing levels well above 35 dB, which is not the case in the “discordant profile” presented by these CD1 mice.

This profile is associated with a tonotopic disorder, revealed by a large shift in the best masking frequency of MTC towards the low frequencies when the probe tone-burst is set within the interval with discordant DPOAE versus ABR thresholds. A possible pitfall of the MTC technique relates to the presence of a transient low-frequency artefact sound in addition to the high-frequency stimulus, which if intense enough would produce a spurious ABR response from the still normally sensitive apical part of the cochlea. However, the envelope of the probe tone-burst was a Blackman-Harris window that optimally softens the rising and falling transients driving the earphone. Although tone-bursts at and above 20 kHz did present a secondary low-frequency artefact around 10–12 kHz, its amplitude was more than 45 dB below the main stimulus peak. Thus, with a high-frequency probe level <65 dB SPL for plotting a MTC, this artefact is too low for the apical cochlea, even when still normally sensitive, to respond.

A normal masking curve is made of two parts, a sharp tip centered on the probe frequency at which masking is obtained for the lowest masker level and a broad tail at lower frequencies, at which maskers about 40–50 dB above probe level can still exert efficient masking. Masking occurs when action potentials produced by a probe tone just above its detection threshold, which activates only the auditory neurons most sensitive to it, are swamped by the activity produced in the same neurons by a masker sound at another frequency. In a masking experiment, the ability to respond to the probe or masker sounds is not an intrinsic property of the neurons, but of the inner hair cells to which the responding neurons are connected. Ultimately, inner hair cell responses passively reflect the local cochlear micromechanics chiefly determined by OHCs (e.g., [25]) and so does masking. Caveats to this rule, discussed later, are that in the presence of either of the regions with dead neurons or of leaking propagation channels that would bypass the basilar membrane [14], masking may be determined, not by OHC status at the place tuned to the probe tone, but rather, at the off-resonance place where neuronal responses to the probe are generated. Hence, the fact that a fraction of CD1 cochlear neurons may have suffered damage at the tested stage could only influence the size of neuronal responses to probe or masker tones, not the frequency dependence of the masking phenomenon itself.

Hypersensitive tails of a masking curve, one possible description of the shifted masking curves observed here, have received first explanation from the study of Liberman and Dodds [25] where single unit tuning curves presenting a hypersensitive low-frequency region were reported, particularly from neurons coming from cochlear places with OHCs disconnected from the TM. A systematic description of the phenomenon on the single unit level was performed in noise exposed guinea pigs [26]. While the loss of the tip reflects the loss of active resonance of the cochlear amplifier, when OHCs no longer exert amplification, increased sensitivity to masking at low frequencies is attributed to the increased mobility of the TM, due to loss of coupling with the organ of Corti in absence of OHC [27] that confers to the cochlear base an increased sensitivity to low-frequency maskers. In less severe circumstance, where OHCs are damaged but present, the MTC tip is located at the characteristic frequency and an increase in tail sensitivity is also visible [26].

The “discordant profile” of CD1 mice does not correspond to already described models of hypersensitive masking tail, in that the masking of high-frequency probes by low-frequency maskers is greater with MTCs forming a marked drop, down to levels near the probe level (Figure 3(c)). In MTC with standard “hypersensitivity of the tail,” the masking effect occurs at a level only about 20 dB lower than in a normal MTC. The “discordant profile” is thus more reminiscent of “dead regions,” where the neuronal response to stimuli at frequencies corresponding to these zones in which inner hair cells or neurons are totally lost is shifted to functional adjacent areas [28]. Thus, the MTCs obtained in this case have their tip at a frequency corresponding to the nearest functional region, as it is from this region that neuronal responses to the probe come. The stimulus must be of sufficient intensity for the vibration to propagate to the functional area. In the case of the CD1 “discordant profile,” it seems difficult, however, to assume that a probe stimulus at 32 kHz and at about 65 dB SPL would be sufficient to produce a response of the place tuned to the MTC tip, around 12 kHz. The “discordant profile” of CD1 seems correlated with a presence of basal OHC whose stereociliary bundles sometimes exhibit abnormal conformations, as it tends to disappear once significant OHC losses appear (Figure 3(c) at P25). A coupling persists between these disorganized stereociliary bundles and the overhanging TM, as evidenced by the imprints found on its inferior side (Figure 7(h)). This profile resembles the recently reported* Nherf1*^−/−^ mice profile, which present shifted MTC tips and extant yet totally nonfunctional basal OHCs with deeply altered hair bundles. The hypothesis formulated in the study of* Nherf1*^−/−^ mice was that the tip of MTCs revealed that the responses to high-frequency probes actually came from more apical places than allowed by the normal tonotopy. Propagation of sound waves along the basilar membrane, as what happens in the case of basal dead regions, would have been too attenuated to allow deep tips to be observed and it was proposed that propagation occurred along the TM in relation to its persistent coupling with nonfunctional OHCs, able to leak vibrations without filtering them. Whereas the* Nherf1*^−/−^ profile is caused by the genetic lack of one molecule of the hair bundle, resulting in targeted alterations of the OHC stereocilia, the “discordant profiles” found in the outbred strain CD1 do not occur in all mice, whose degrees of hearing loss and patterns of stereocilia defects are highly variable and occur in variable degrees (Figures 3(a) and 3(c)). Indeed, from P25 to 32 kHz, this profile is replaced by a more traditional sensorineural hearing loss. Of course, one cannot exclude the fact that the “discordant profiles” of CD1 mice represent a form of extreme “hypersensitivity of the tail,” with the place responding to high-frequency probe tones being still at its normal tonotopic place. The unusual intensity with which distorted modes of vibration occur in response to lower frequency maskers would be due to the abnormal basal mechanics produced by a peculiar coupling between the TM and abnormal OHCs.

Some CD1 mice presented a less pure “discordant profile” than* Nherf1*^−/−^ mice with the presence of residual DPOAE at 22 kHz (Figure 5). A persistent ability to generate small DPOAEs, absent in* Nherf1*^−/−^ mice and in CD1 mice at 32 kHz, does not preclude that coupling with the TM might be at the origin of a perturbed tonotopy as hypothesized above.

Irrespective of the explanatory mechanics, the expected perceptive consequences of a “discordant” functional pattern as described here are a rather better sensitivity to high frequencies than the one predicted with totally nonfunctional OHCs, allowing a sort of off-frequency listening to occur. As a counterpart, the “better than expected” ability to detect high frequencies, with an optimistic pure-tone audiogram that does not attract the clinician's attention, comes with no guarantee that suprathreshold behavior is normal, with the additional penalty of increased sensitivity to low-frequency masking. In clinical studies, it has been reported that minimal losses on high frequencies (≤30 dB), observed in some subjects, have deleterious effects on speech perception, normally associated with low or medium frequencies [15]. This decrease in intelligibility could result from suprathreshold deficiencies caused by OHC damage. Recently, another study found a correlation between loss of OHC function and reduced speech-in-noise performance, in subjects with minimal high-frequency hearing loss [16]. The “discordant profile” presented by some CD1 mice may therefore correspond to a reality in some patients, complaining of impaired intelligibility, but with normal audiometric thresholds and no neural alteration.

In conclusion, although it has been rightly emphasized that a clinical pattern with difficulties in the presence of noise out of proportion with the pure-tone audiogram should raise the possible diagnosis of auditory-neuropathy spectrum disorder [11], such difficulties may also be the hallmark of abnormal OHC function, when atypical OHC lesions occur [17], which the present work confirms, moreover, in an animal model that is thought to be a good model of the most frequent cause for sensorineural hearing loss, presbycusis [24]. An easy means for separating the two frameworks lies beyond pure-tone audiometry, with a contrast between extant OAEs and distorted ABRs in the case of neuropathy and absent OAEs and normal ABRs, in the case of “discordant profile.”

## Abbreviations

ABR: Auditory brainstem response
CM: Cochlear microphonic
DPOAE: Distortion product otoacoustic emission
ENT: Ear, nose, and throat
FEG: Field emission gun
HL: Hearing loss
HMDS: Hexamethyldisilazane
IHC: Inner hair cell
MTC: Masking tuning curve
OAE: Otoacoustic emission
OHC: Outer hair cell
PBS: Phosphate buffer saline
PFA: Paraformaldehyde
PHEM: Pipes, Hepes, Egtazic acid, and Magnesium
SNHL: Sensorineural hearing loss
SPL: Sound pressure level
TM: Tectorial membrane.

## Additional Points

*Highlights*. (i) In CD1 mice, abnormal OHC function can come with near-normal auditory thresholds. (ii) The discrepancy occurs where hair cell to tectorial-membrane connections are abnormal. (iii) Low frequency masking of the high frequencies is excessive in CD1. (iv) Performance at threshold hides suprathreshold auditory impairment.
